# Supplementary material for: Decadal oscillation provides skillful multiyear predictions of Antarctic sea ice
Source: Nat Commun. 2023 Dec 13;14:8286. doi: 10.1038/s41467-023-44094-1 (PMC10719290; doi:10.1038/s41467-023-44094-1)
Supplement: Supplementary file 1 — Supplementary material [file 41467_2023_44094_MOESM1_ESM.pdf]

Supplementary Method:

Indo-Pacific SST-forced experiment: We conduct an experiment where the model is prescribed with a significantly extended range within 45°S–45°N and 30°E–70°W, which covers the whole Indo-Pacific SST region at lower latitudes to test the model's capability to reproduce the realistic sea ice variability. The experiment is conducted from 1955 to 2022, and the spin-up time is consistent with that configured in the CP_EXP. Considering that the decadal variance (8-16 years) of the Atlantic SST is comparably weak (Fig. 3a in the manuscript), its influence on the model performance in simulating the decadal oscillation in Antarctic sea ice may be negligible. Here we mainly focus on the Indo-Pacific region, which contains primary decadal signals of the global SST in the observation.

The correlation map between the PQDO index and SAT/geopotential height shows a consistent pattern as that in the observation. In Supplementary Fig. 6a, heating in the central tropical Pacific excites a poleward propagating Rossby wave train. The centers of geopotential height correspond well with the observation, especially the weakened Amundsen Sea Low downstream of this wave train, suggesting that the coupled model is able to reproduce the observed tropical-polar teleconnection. The SAT warming signal is located east of the weakened ASL, indicating that the local mechanism related to temperature advection and shortwave radiation plays a role. This experiment also successfully reproduces the reversed relationship between the sea ice and the tropical SST forcing. The SIC over the Ross Sea and the Amundsen Sea declined significantly in response to the PQDO in the Indo-Pacific experiment, and the spatial patterns overall agreed with the observation, despite that its intensity is slightly underestimated (Supplementary Fig. 6b).

More importantly, the Indo-Pacific SST-forced experiment captures the low-frequency variability in the atmosphere-sea ice coupled system over the Ross-Amundsen Seas. As shown in Supplementary Fig. 6c, the series of Ross-Amundsen Seas SIC exhibits a

significant decadal oscillation, with a spectral peak within 8–16 years (Supplementary Fig. 6d). The simulated Ross-Amundsen Seas SIC has consistent phases and peaks as in the observation. The SIC is well correlated with the ASL and local SAT (r = -0.75 and -0.97, respectively), which exhibit a synchronized quasi-periodicity (Supplementary Fig. 6e). The above results suggest that the coupled model has the capability to simulate the decadal oscillation in Antarctic sea ice and the associated local variables, as well as the tropical-polar teleconnection.

Further, we can compare the results between the CP_EXP and Indo-Pacific SST-forced experiment. Despite the significant differences that can be found in the locations of ASL, the SIC averaged over the Ross-Amundsen Seas shows consistent decadal fluctuations. The Ross-Amundsen Seas SIC simulated in CP_EXP explained over 60% quasi-decadal (8–16-yr) variance of that in the Indo-Pacific SST-forced experiment, further suggesting that the central tropical Pacific SST is the primary source of decadal variability of the Ross-Amundsen Seas SIC. We must note that the model biases can be reduced with more observational signals being introduced into the experiment. Thus, it is reasonable that the SIC pattern in the Indo-Pacific SST-forced experiment seems closer to the observed pattern. For example, the climatology of Antarctic SIC in the Indo-Pacific SST-forced experiment shows slightly reduced high biases compared to the CP_EXP (Supplementary Fig. 6f and 6g), despite that biases still exist compared to the observation.

Supplementary Figures:

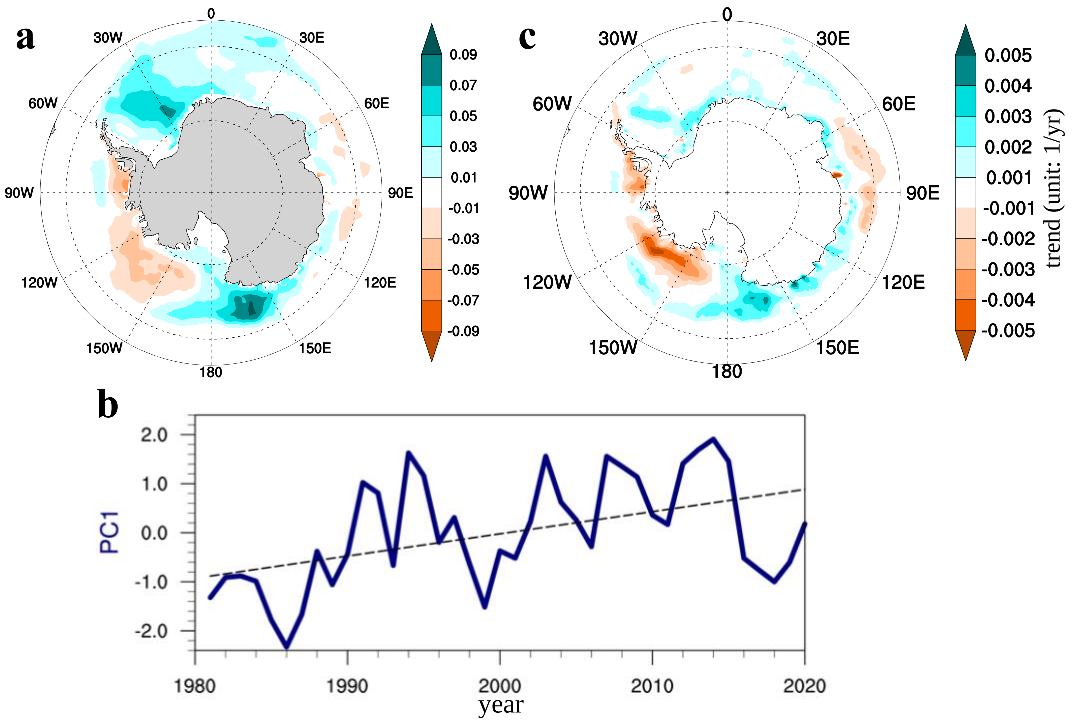

Supplementary Fig. 1 (**a**) The EOF1 of Antarctic sea ice concentration. (**b**) The PC1 of Antarctic sea ice concentration. The black dashed line indicates the long-term trend. The EOF is computed pan-Antarctic within 50°S–90°S. (**c**) The trend pattern of Antarctic sea ice concentration (unit: 1/yr) for the period 1981–2020.

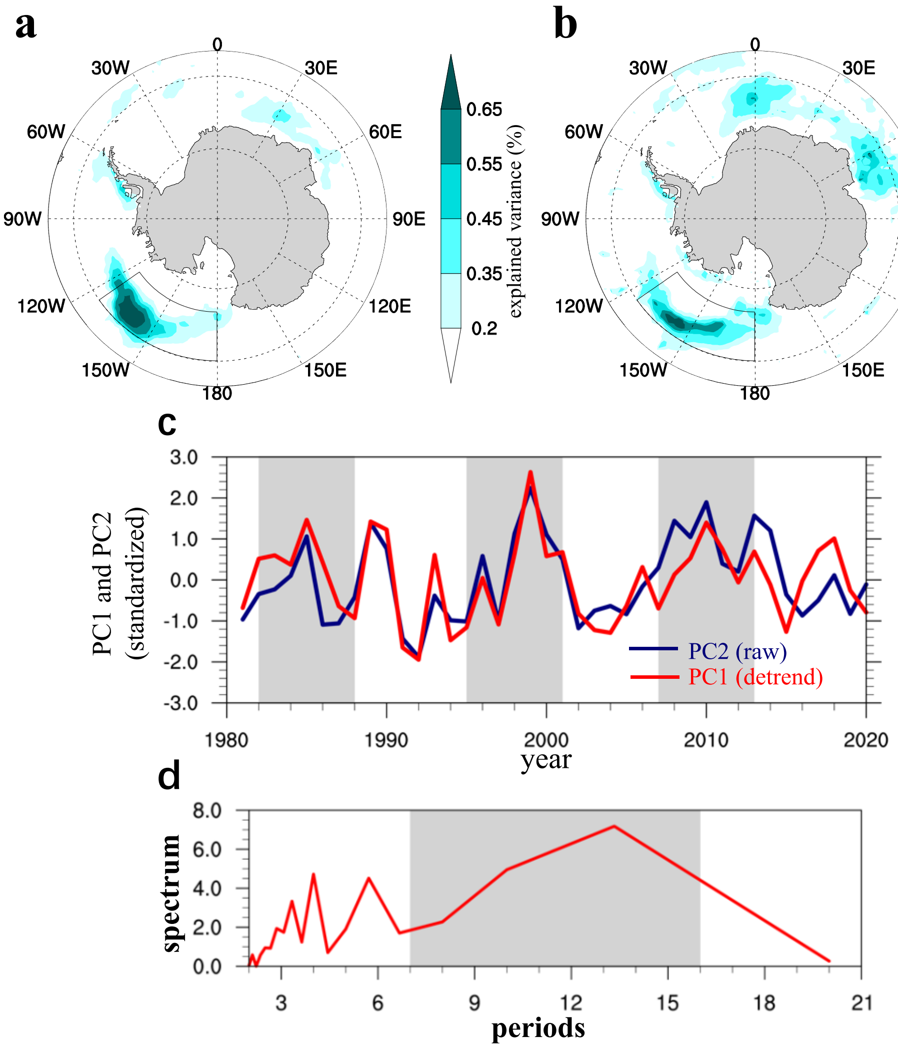

Supplementary Fig. 2 (**a**) The explained variance of the PC2 on Antarctic sea ice (raw data); (**b**) as in (**a**), but for 8-yr lowpass filtered data. (**c**) The PC1 of the detrended annual Antarctic SIC (red line) and the PC2 of the raw annual Antarctic SIC (blue line); (**d**) The power spectrum of the PC1 of the detrended annual Antarctic SIC

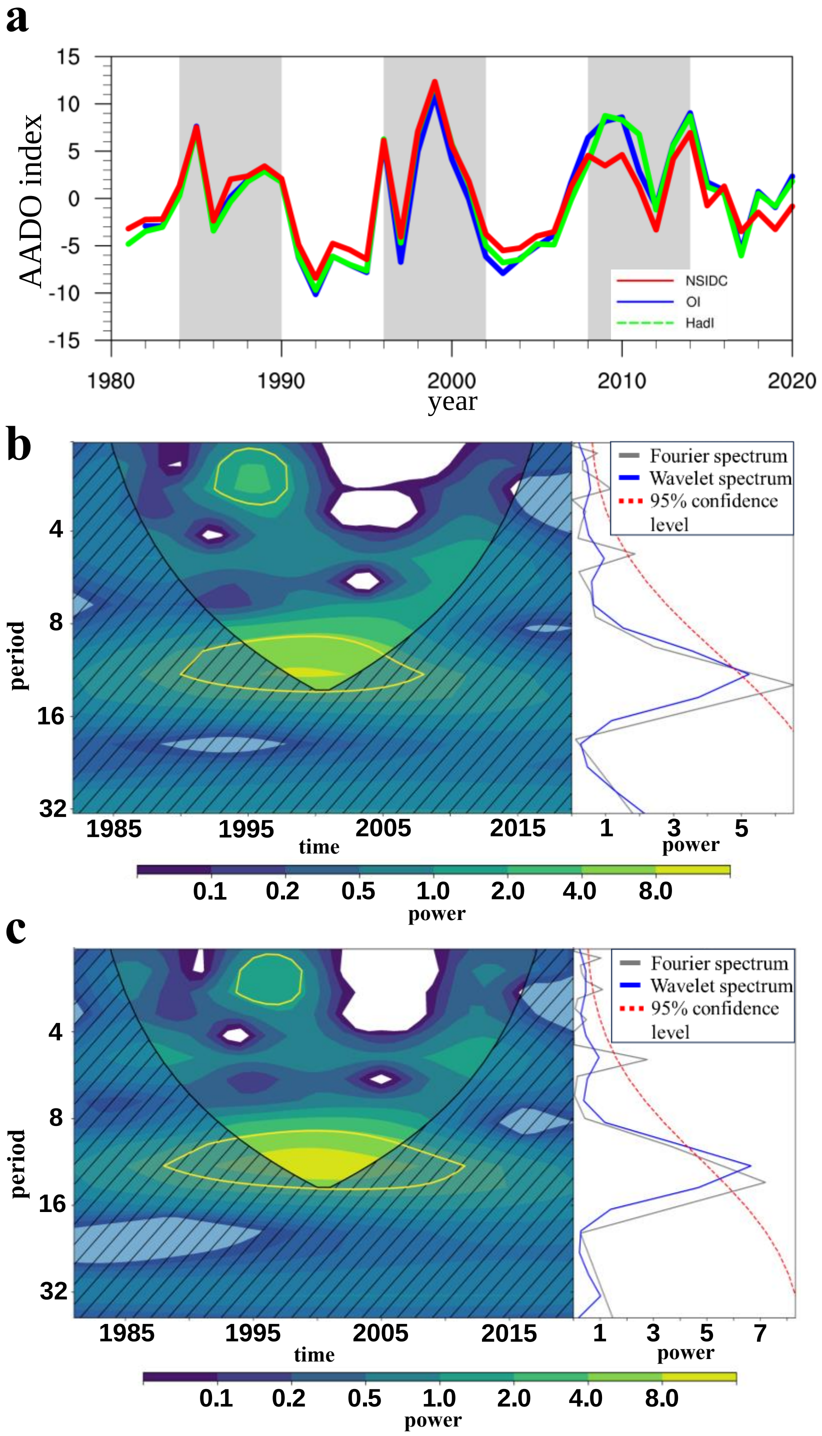

Supplementary Fig. 3 (**a**) The detrended and normalized time series of the AADO index derived from the NSIDC (red), OISST (blue), and HadISST (green) datasets. The OISST started in 1982, while the other two data started in 1981. (**b**) and (**c**) are the local wavelet power spectrums and global wavelet/Fourier spectrums for the AADO index using the OISST and HadISST, respectively. The yellow contour indicates the 95% significance level using a red-noise background spectrum. The red dash line in the right-hand panel indicates the 95% confidence level for the global wavelet spectrum.

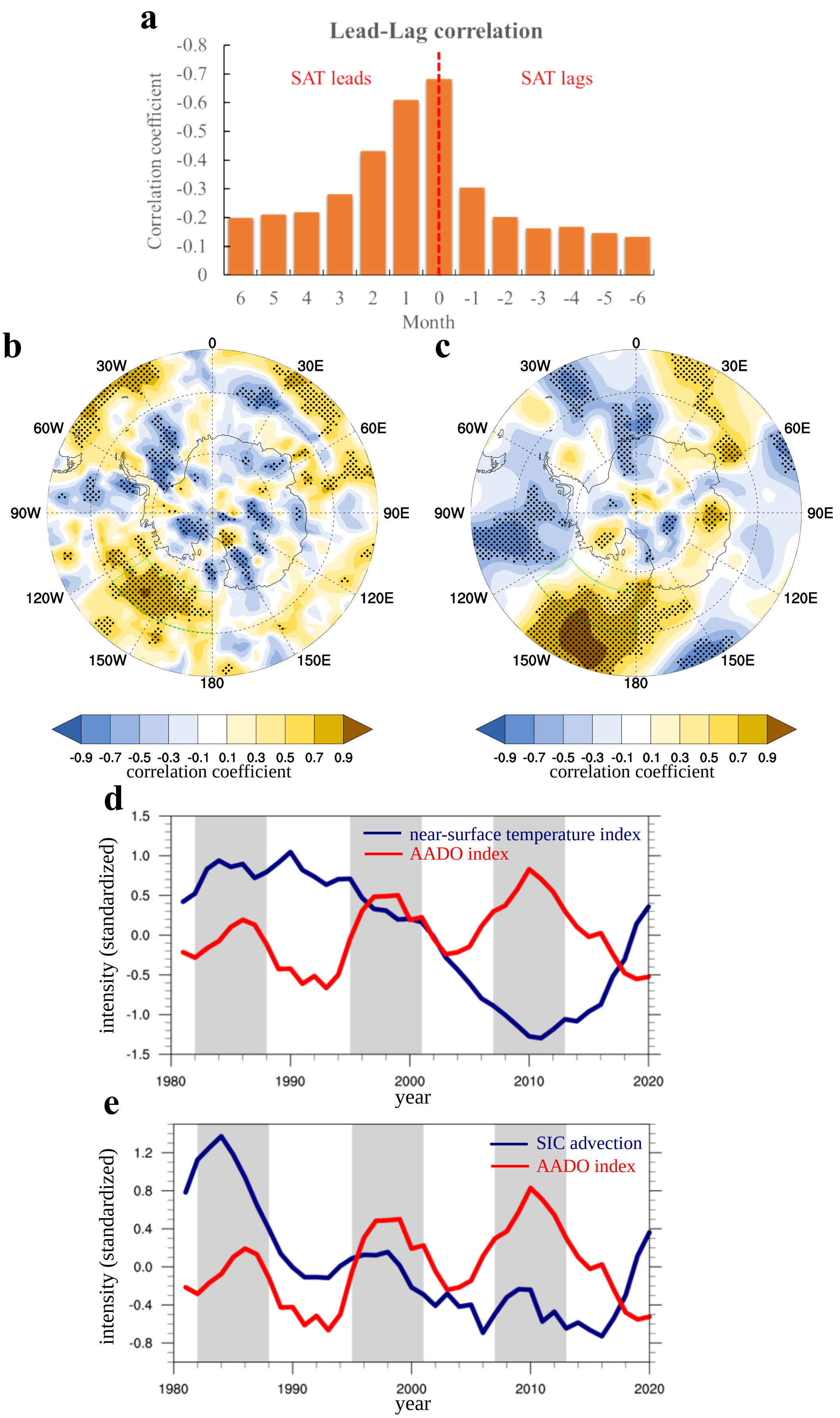

a
Lead-Lag correlation
SAT leads
SAT lags
Correlation coefficient
-0.8
-0.7
-0.6
-0.5
-0.4
-0.3
-0.2
-0.1
0
6 5 4 3 2 1 0 -1 -2 -3 -4 -5 -6
Month
b
c
0
30W
30E
60W
60E
90W
90E
120W
120E
150W
150E
180
-0.9 -0.7 -0.5 -0.3 -0.1 0.1 0.3 0.5 0.7 0.9
correlation coefficient
d
near-surface temperature index
AADO index
intensity (standardized)
1.5
1.0
0.5
0.0
-0.5
-1.0
-1.5
1980 1990 2000 2010 2020
year
e
SIC advection
AADO index
intensity (standardized)
1.2
0.8
0.4
0.0
-0.4
-0.8
1980 1990 2000 2010 2020
year

Supplementary Fig. 4 (**a**) The lead-lag correlation between the AADO index and the SAT index, which is calculated from the monthly data. The x-axis denotes the lead months of SAT. The correlation map of the ASL index (150°W–90°W, 60°S–70°S) with (**b**) shortwave radiation and (**c**) meridional temperature advection at 850 hPa. The data has been preprocessed by an 8–16-yr bandpass filter. The dotted shading indicates the correlation coefficient is significant at the 95% confidence level. The normalized and 8-yr lowpass filtered time series of the AADO index (red line) and the Ross-Amundsen Seas (**d**) near-surface ocean temperature at 25m and (**e**) SIC advection (both are blue lines).

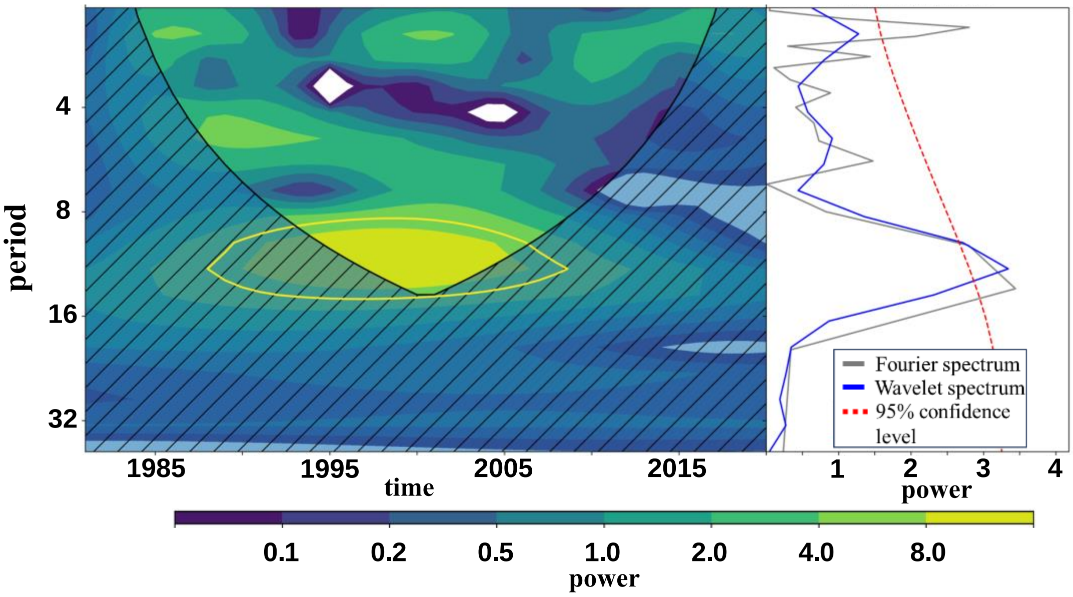

Supplementary Fig. 5 The local wavelet power spectrums and global wavelet/Fourier spectrums of the Amundsen Sea Low. The yellow contour indicates the 95% significance level using a red-noise background spectrum. The red dash line in the right-hand panel indicates the 95% confidence level for the global wavelet spectrum.

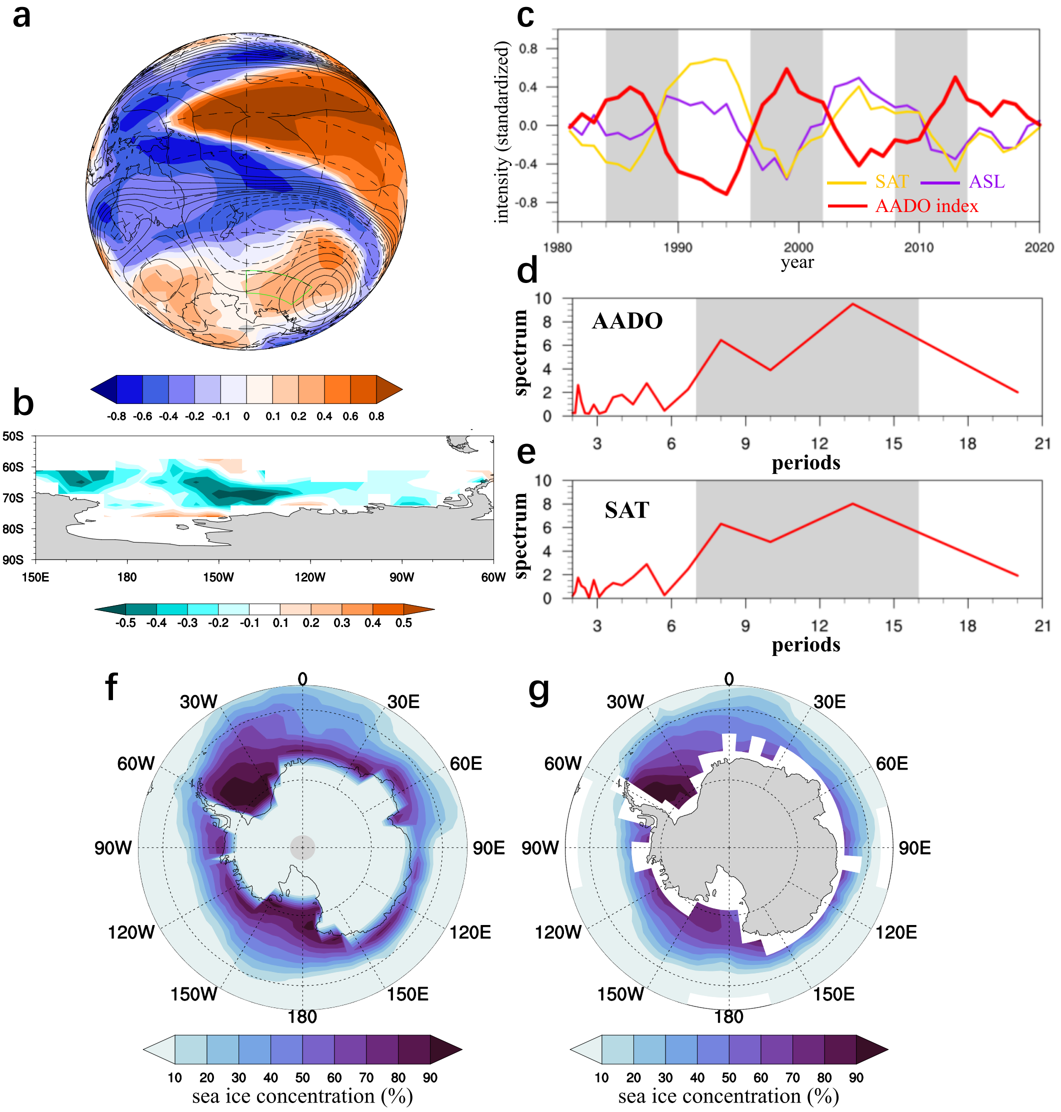

Supplementary Fig. 6 (**a**) The correlation map of the PQDO index with the SAT (shading) and geopotential height (contours) at 200hPa in the Indo-Pacific SST-forced experiment (the description is shown below); (**b**) The correlation between the PQDO index and the SIC over the Ross-Amundsen Seas; (**c**) The normalized series of the AADO index, the ASL index, and the SAT averaged over the Ross-Amundsen Seas. All data used here is processed by an 8-yr lowpass filter; The power spectrums of (**e**) SAT and (**d**) SIC over the Ross-Amundsen Seas, respectively. The climatology of Antarctic SIC (unit: %) in (**f**) the Indo-Pacific SST-forced experiment and (**g**) the observation, respectively. The climatology is computed for the period 1981–2020.

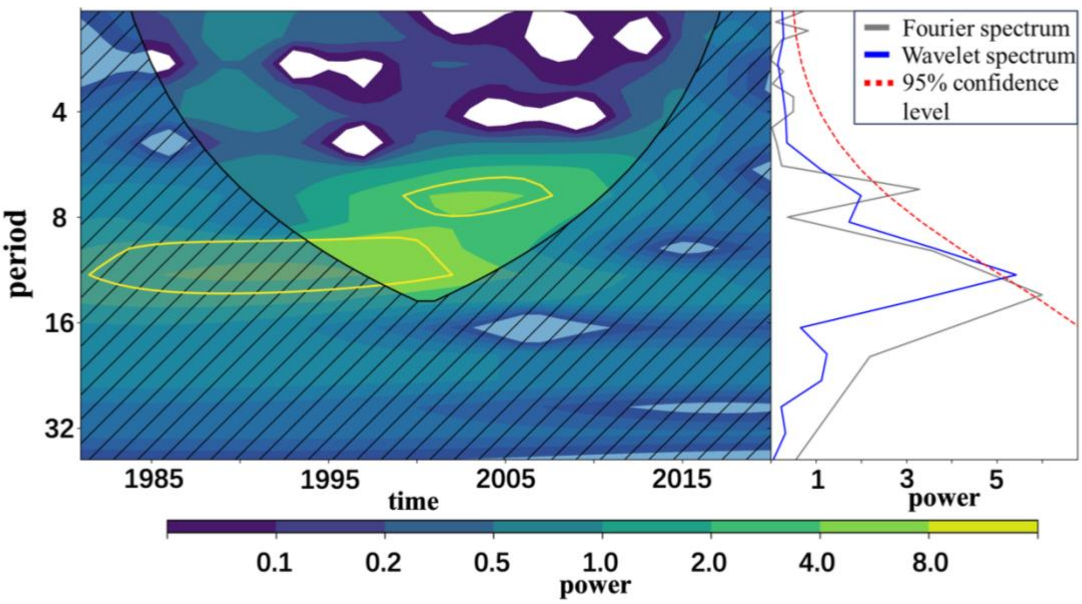

Supplementary Fig. 7 The local wavelet power spectrums and global wavelet/Fourier spectrums of the AADO simulated in CP_EXP. The yellow contour indicates the 95% significance level using a red-noise background spectrum. The red dash line in the right-hand panel indicates the 95% confidence level for the global wavelet spectrum.

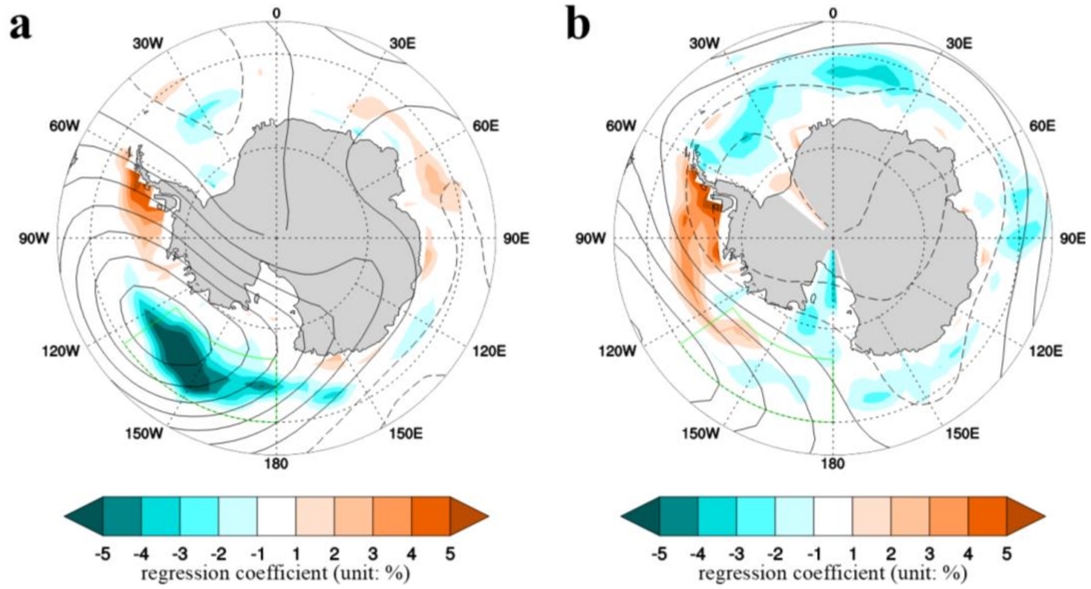

Supplementary Fig. 8 The composited regression maps of Antarctic SIC (shading; unit: %) and 200 hPa geopotential height (contour; unit: m) onto the PQDO index in the (**a**) Group 1 and (**b**) Group 2. The data is analyzed for the period 1981–2014.

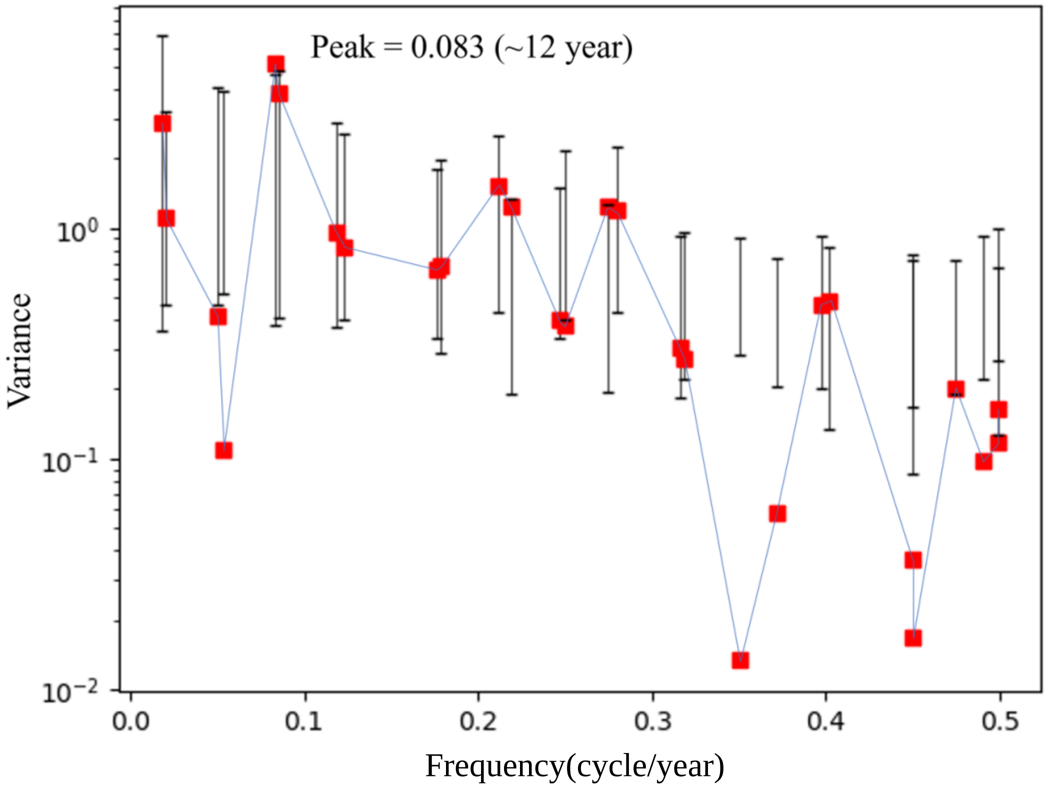

Supplementary Fig. 9. The power spectrum of the observed PQDO index for an extended period 1960–2020. The significance of the spectrum peak is tested using the Monte Carlo Singular Spectrum Analysis (see Method), with 5000 realizations. The error bar indicates the 95% confidence level of the spectrum. The red dots appear well above the error bars, indicating that the signals they contain in the data series are statistically significant.

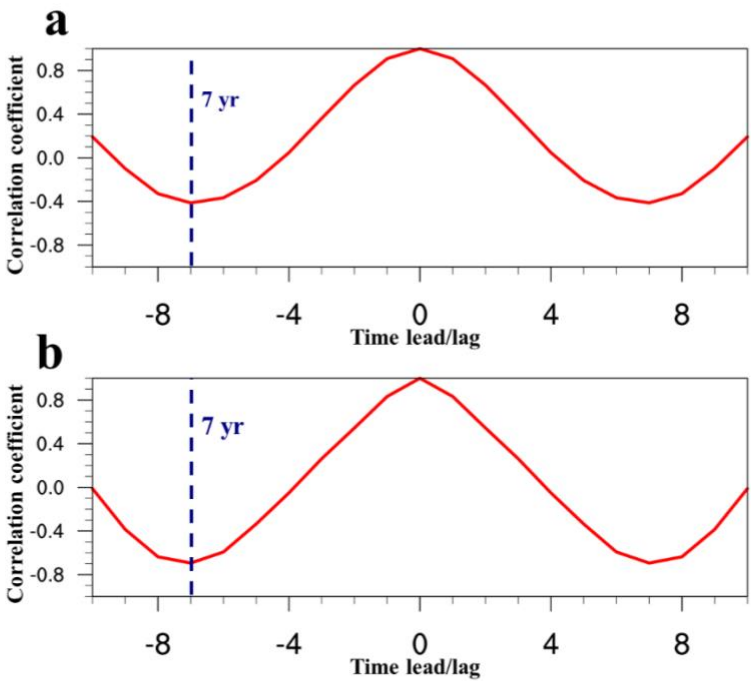

Supplementary Fig. 10 The lead-lag auto-correlation of the PQDO index for the period (**a**) 1981–2020 and (**b**) 1981–2009. The PQDO index has been preprocessed by 8-yr running mean to isolate the decadal component. The PQDO shows significant autocorrelation when it leads/lags by seven years, and the result is independent of the analyzed period.

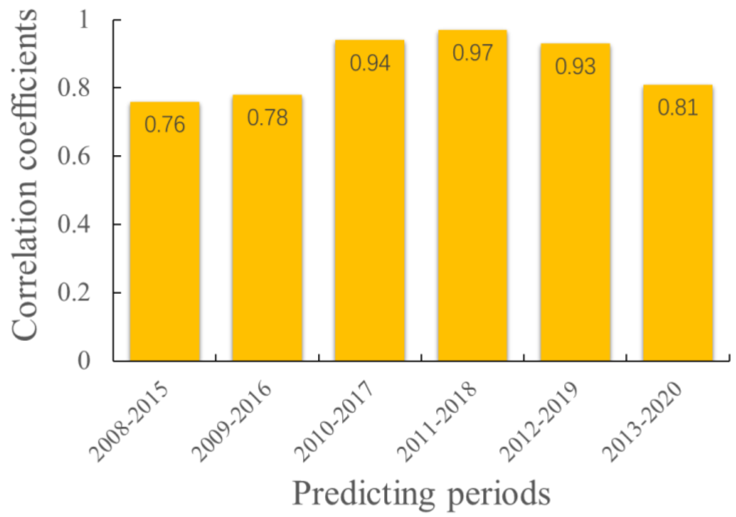

Supplementary Fig. 11 The correlation coefficients between the observed and predicted AADO index during the six predicting periods.

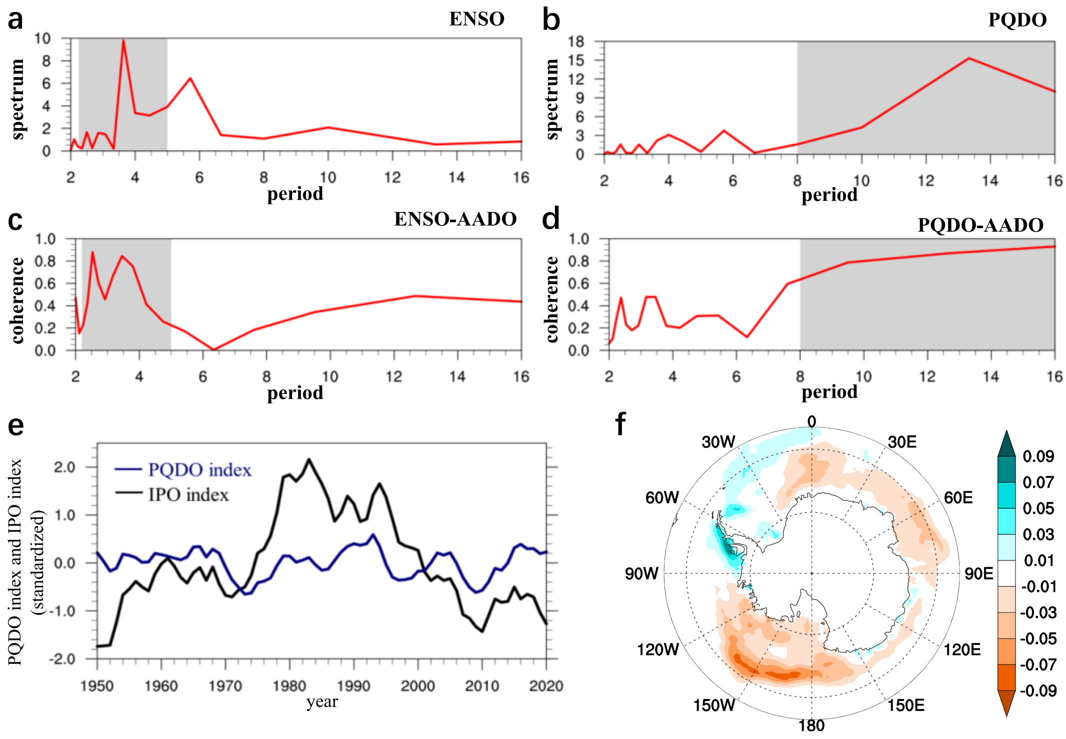

Supplementary Fig. 12 The power spectrums of (**a**) ENSO (indicated by the Nino 3 index, and hereafter), (**b**) the PQDO index. The spectral coherence of the AADO with (**c**) ENSO and (**d**) the PQDO index. (**e**) The normalized series of the IPO index and the PQDO index (8-yr lowpass filtered) for the period 1950–2020. (**f**) The regression of Antarctic SIC onto the 8-yr lowpass filtered PQDO index (unit: 1) after removing the IPO signal. The IPO signal is removed by subtracting the IPO-regressed SIC from the raw data. The residual is then regressed onto the PQDO index.

Supplementary Tables:

Supplementary Table. 1 CMIP6 models

No.	Model	Country/region	Resolution
1	NorESM2-MM	Norway	1° × 1°
2	CMCC-ESM2	Italy	0.9° × 1.25°
3	NESM3	China	1.9° × 1.9°
4	TaiESM1	Taiwan	0.9° × 1.25°
5	CMCC-CM2-SR5	Italy	1° × 1°
6	MPI-ESM1-2-LR	German	1.5° × 1.5°
7	NorESM2-LM	Norway	2.5° × 2°
8	FIO-ESM-2-0	China	1.25° × 0.9°
9	ACCESS-CM2	Australia	1.875° × 1.25°
10	MRI-ESM2-0	Japan	1° × 0.5°
11	CESM2-WACCM	United States	0.9° × 1.25°
12	IPSL-CM6A-LR	France	2.5° × 1.3°
13	E3SM-1-1	United States	1° × 1°
14	GFDL-ESM4	United States	1.3° × 1°
15	HadGEM3-GC31-LL	UK	1° × 1°
16	E3SM-1-0	United States	1° × 1°
17	MPI-ESM1-2-HR	German	0.94° × 0.94°
18	EC-Earth3-Veg-LR	Europe	1° × 1°
19	BCC-CSM2-MR	China	1.125° × 1.125°
20	EC-Earth3-CC	Europe	1° × 1°
21	CAS-ESM2-0	China	1.4° × 1.4°
22	EC-Earth3-Veg	Europe	0.7° × 0.7°
23	INM-CM5-0	Russia	2° × 1.5°
24	E3SM-1-1-ECA	United States	1° × 1°
25	CAMS-CSM1-0	China	1.125° × 1.125°
26	CIESM	China	1° × 1°
27	FGOALS-g3	China	2° × 2.25°
28	ACCESS-ESM1-5	Australia	1.875° × 1.25°
29	UKESM1-0-LL	UK	1.9° × 1.3°

The criterions selecting models into Group 1 and Group 2:

In this section, we tend to inspect whether the AADO and its teleconnection to the PQDO can be captured by CMIP6 models. The premise is that models can reproduce the PQDO to some extent, with the quasi-decadal variance ratio of the central tropical Pacific SST greater than 14%. Then, the selection of models for Group 1 and Group 2 synthetically considers the quasi-decadal variance ratio of SIC and the correlation between PQDO and AADO. Scoring metrics based on the correlation coefficients of PQDO-AADO are employed for models that are capable (or incapable) of reproducing the variance ratio in SIC.

First, we need to compare the reproducibility of the relationship between PQDO and AADO among models. The scoring metric is defined as follows:

$$score(i) = 0.7 * [-R_{raw}(i)] + 0.3 * [-R_{bandpass}(i)]$$

where the score of the model (i) is a weighted combination of the correlation coefficients between PQDO and AADO using raw and 8–16-yr bandpass filtered data. Despite that the filtered data highlights the decadal components, the raw series contains more signal that is important to comprehensively evaluate their coherence. The scores are listed from largest to smallest in Supplementary Table. 2. For models with lower scores, the simulated relationships between PQDO and AADO are relatively weak, which are sorted into Group 2. On the other hand, the NorESM2-LM, NorESM2-MM, CESM2-WACCM, CMCC-ESM2, and FIO-ESM-2-0 are the top five models with higher scores, indicating that the PQDO-AADO teleconnection is relatively well reproduced. Additionally, models with high scores should also be capable of capturing the low-frequency signals in the Ross-Amundsen Seas SIC, which requires a decadal variance ratio greater than 10%. Thus, only NorESM2-LM, NorESM2-MM, CMCC-ESM2, and FIO-ESM-2-0 are selected for Group 1.

Supplementary Table. 2 Scores

Model	Variance ratio of the AADO	Score	Note
NorESM2-LM	16.38	0.814	Group 1
NorESM2-MM	34.34	0.795	Group 1
**Observation**	**42.22**	**0.68**	
CESM2-WACCM	8.87	0.665	
CMCC-ESM2	27.05	0.615	Group 1
FIO-ESM-2-0	13.42	0.582	Group 1
TaiESM1	16.49	0.576	
CMCC-CM2-SR5	15.57	0.511	
IPSL-CM6A-LR	25.72	0.446	
MRI-ESM2-0	7.8	0.247	Group 2
ACCESS-CM2	6.75	0.217	Group 2
NESM3	7.92	0.206	Group 2
MPI-ESM1-2-LR	7.15	-0.015	Group 2
